# Effect of intensivist involvement on clinical outcomes in patients with advanced lung cancer admitted to the intensive care unit

**DOI:** 10.1371/journal.pone.0210951

**Published:** 2019-02-13

**Authors:** Jin Hwa Song, Sooyeon Kim, Hyun Woo Lee, Yeon Joo Lee, Mi-jung Kim, Jong Sun Park, Yu Jung Kim, Ho Il Yoon, Jae Ho Lee, Jong Seok Lee, Choon-Taek Lee, Young-Jae Cho

**Affiliations:** 1 Division of Pulmonary and Critical Care Medicine, Department of Internal Medicine, Seoul National University Hospital, Ihwa-dong, Jongno-gu, Seoul, South Korea; 2 Medical Research Collaborating Center, Seoul National University Bundang Hospital, Bundang-gu, Seongnam, South Korea; 3 Division of Pulmonary and Critical Care Medicine, Department of Internal Medicine, Seoul National University College of Medicine, Seoul National University Bundang Hospital, Bundang-gu, Seongnam, South Korea; 4 Medical Oncology, International St. Mary's Hospital, College of Medicine, Catholic Kwandong University,Seo-gu, Incheon, South Korea; 5 Division of Hematology and Medical Oncology, Department of Internal Medicine, Seoul National University College of Medicine, Seoul National University Bundang Hospital, Bundang-gu, Seongnam, South Korea; University of Notre Dame Australia, AUSTRALIA

## Abstract

**Purpose:**

Intensive care unit (ICU)-related mortality for lung cancer is ranked highest among the solid tumors and little information exists on the role of intensivists on clinical outcomes. This study aimed to elucidate the intensivist’s contribution toward clinical outcomes.

**Materials and methods:**

Data of advanced lung cancer patients, including stage IIIB or IV non-small cell lung cancer and extensive-stage small cell lung cancer, admitted to the ICU from 2005 to 2016 were analyzed. Multivariate logistic regression was performed to determine variables associated with ICU and in-hospital mortality. Autoregressive integrated moving average (ARIMA) for time-series was used to assess the intensivist’s impact.

**Results:**

Of 264 patients, 85 (32.2%) were admitted to the ICU before and 179 (67.8%) after organized intensive care introduction in 2011. Before and after 2011, the changes observed were as follows: ICU mortality rate, 43.5% to 40.2%, respectively (p = 0.610); hospital mortality rate, 82.4% to 65. 9% (p = 0.006). The duration of ICU and hospital stay decreased after 2011 (14.5±16.5 vs. 8.3 ± 8.6, p < 0.001; 36.6 ± 37.2 vs. 22.0 ± 19.6, p < 0.001). On multivariate analysis, admission after 2011 was independently associated with decreased hospital mortality (Odds ratio 0.42, 95% confidence interval 0.21–0.77, p = 0.006). In ARIMA models, intensivist involvement was associated with significantly reduced hospital mortality. (Estimate -17.95, standard error 5.31, p = 0.001)

**Conclusion:**

In patients with advanced lung cancer, organized intensive care could contribute to improved clinical outcomes.

## Introduction

Lung cancer is the leading cause of cancer death in South Korea [1] and worldwide [2]. Moreover, it is the most common cause of intensive care unit (ICU) admission among solid tumors, and the number of admissions has increased over time in the United States [3, 4]. The critical illness in lung cancer patients is mainly associated with respiratory dysfunction due to multiple reasons: 1) cancer-related complications, such as airway obstruction or bleeding, pulmonary embolism, superior vena cava syndrome, and neurologic problems; 2) treatment-related complications, such as radiation pneumonitis and anti-tumor drug-induced interstitial pneumonia; and 3) infections, especially obstructive pneumonia [5]. Patients with lung cancer often require intensive care due to the aggressive nature of the disease.

Although survival in critically ill patients with cancer has improved over the decades [6, 7], ICU mortality related to lung cancer is ranked highest among the solid tumors [8]. In a multi-national study published in 2014, which included a high percentage of newly-diagnosed patients (71%), lung cancer patients had a high rate of ICU mortality (29%) [9].

There has been a continuing discussion regarding ICU admission criteria for cancer patients [10, 11], and oncologists and intensivists have different views in this regard [12]. Recent advances in immunotherapy and targeted therapy have led many experts to believe that the prognosis of lung cancer is likely to improve dramatically [13]. Therefore, it is important to renew the discussion about how lung cancer patients should receive intensive care and treatment.

In a previous study conducted in our hospital [14], we analyzed the clinical status of advanced lung cancer patients admitted to the medical ICU and categorized patients according to the guidelines outlined by Darmon et al [11]. According to this study, refractory disease and poor performance status were related to worse ICU outcomes. Since 2011, our center has provided organized intensive care services by board-certified intensivists. Although many studies have reported outcomes related to introducing intensive care specialists [15, 16], there is no study describing the influence of the intensivist system on critically-ill patients with advanced lung cancer.

The objective of our study was to evaluate the effect of involvement of the pulmonary intensivist on clinical outcomes in advanced lung cancer patients and to investigate clinical factors associated with ICU mortality in these patients.

## Materials and methods

### Study population

Lung cancer patients with histopathologically proven non-small cell lung cancer (NSCLC) (stage IIIB or stage IV) and extensive stage (ED) small cell lung cancer (SCLC) were included in the study; we defined this group as advanced lung cancer. From 2005 to 2016, 264 advanced lung cancer patients older than 18 years were admitted to the medical ICU in a single-center, tertiary teaching hospital in Korea. We conducted a retrospective review of medical records of these patients. If the patient was admitted several times in the ICU, only the first admission was included in the study.

### Intensivist system implementation

From 2011, our hospital started providing intensive care services by dedicated pulmonology intensivists. After the introduction of the critical care specialist, we admitted patients eligible for intensive care and used a multi-disciplinary approach. Regular education of the ICU house-staff with regard to sepsis management and mechanical ventilation weaning were started. Patients from other departments were also referred to the pulmonary intensivist, and initial clinical decisions were made by the intensivist group. Intensivist referral included change of the primary decision-making physician from the hemato-oncologist to the pulmonary intensivist, and intensivist team guided critical care. We also launched the rapid response team (RRT) in October 2012 [17]. The RRT system, called the “Seoul National University Bundang Hospital (SNUBH) Medical Alert First Responder,” included intensivists and trained nurses. The RRT was activated through the electronic medical record (EMR) screening system when it was confirmed that some criteria out of the 10 established criteria had exceeded the threshold [17, 18]. We also introduced a clinical pharmacist and a dedicated dietitian for intensive care, and included daily team rounds with intensivists, residents, nurses, pharmacists, and nutritional specialists. Documented ARDS and MV weaning protocol were applied and started to be used in clinical practice. (S1 and S2 Appendicies)

### Data collection and processing

We obtained data from the EMR system and records from the RRT. The study design and protocol were approved by the institutional review board of SNUBH (IRB No. B-1708/414-114) and informed consent was waived because of the retrospective nature of the study.

Data with regard to demographic variables (age, gender, smoking history, and co-morbidity) and clinical variables were collected. Categorical variables were described as number (percentages) and continuous variables as mean ± standard deviation. Clinical variables included time since diagnosis, Eastern Cooperative Oncology Group Scale performance status (ECOG PS), cancer status (newly diagnosed, remission/controlled, refractory), pathologic types, TNM stage, and prior history of anticancer therapy. Severity of disease and need for critical care was estimated using the Simplified Acute Physiology Score (SAPS) Ⅱ, the Sequential Organ Failure Assessment (SOFA), and Acute Physiology and Chronic Health Evaluation (APACHEⅡ) score on the first day of ICU admission based on vital signs and laboratory results. Reason for ICU admission was classified based on two methods. The first classification was based on the criteria suggested by Azouley et al.[19] as follows: 1) cancer related events, 2) treatment related events, 3) infection, not clearly related to cancer or treatment, 4) comorbidity related events, and 5) others. The second classification was developed from the viewpoint of the intensivist as follows: 1) respiratory failure, 2) neurologic problems, 3) kidney failure, 4) cardiac dysfunction, and 5) others. Some of the terminology used in the study are defined here: 1) acute respiratory failure was defined as clinical symptoms of respiratory distress, or PaO2/FiO2 <300 mmHg, 2) acute kidney injury was defined as creatinine level >1.4 mg/dL or patients administered continuous renal replacement therapy (CRRT), 3) sepsis was diagnosed according to consensus criteria, 2001 [20]. 4) neutropenic fever was defined as febrile status (body temperature >37.5°C) with absolute neutrophil counts lower than 0.5×10^9^/L. Management in ICU included invasive mechanical ventilation (MV); non-invasive MV, and high flow nasal cannula; CRRT; and use of vasoactive agent, steroids, or chemotherapy. Clinical outcomes of advanced lung cancer patients included 30-day ICU mortality, ICU mortality, in-hospital mortality, ICU length of stay (LOS), and hospital LOS.

#### Statistical analyses

Our primary outcome was the comparison of hospital mortality between advanced lung cancer patients admitted to the ICU before and after the introduction of the intensivist system in 2011. Univariate analyses were performed using Pearson’s chi-square test and Fischer’s exact test for categorical variables and student’s t-test for continuous variables. A nonparametric test for trends based on initial ranks (Wilcoxon rank-sum test, nptrend in STATA) was performed to evaluate the increasing number of patients by year. We considered a two-tailed p value <0.05 as statistically significant and calculated 95% confidence intervals (CIs). To identify factors associated with 30-day ICU mortality or hospital mortality, multivariate logistic regression analysis was conducted. To construct the logistic regression analysis model, epidemiologic data including age and sex were included. Among variables with a p-value < 0.1 in univariate analysis, we selected ECOG PS and SAPS II score for multivariable adjustment of the effect of intensivist system introduction. Finally, we included age, sex, ECOG PS, SAPS II score, and cancer control status in logistic regression model. For the time-series analysis, we used the model of intervention analysis proposed by Box and Tiao.[21] Intervention analysis is an extension of autoregressive integrated moving average (ARlMA) models, developed by Box and Jenkins.[21–24] In this study, we used quarterly time-series of 30-day ICU mortality, overall ICU mortality, hospital mortality, ICU LOS and hospital LOS for developing ARIMA models to assess the implementation of the intensivist system. We used the values from the Akaike Information Criterion and time-series model identification tools such as auto correlation function and partial auto correlation function to identify the appropriate models.[24, 25]

Statistical analyses were performed using SPSS version 22.0 (IBM, Armonk, NY), STATA 13.0 (StataCorp 2013, Stata Statistical Software: Release 13, College Station, TX: StataCorp LP), and R program version 3.3.2 (R Foundation for Statistical Computing). Bar graphs were plotted with GraphPad Prism 5.01 (Graphpad software Inc, San Diego, CA)

## Results

### Annual change in advanced lung cancer patients admitted to the ICU

**Fig 1:**
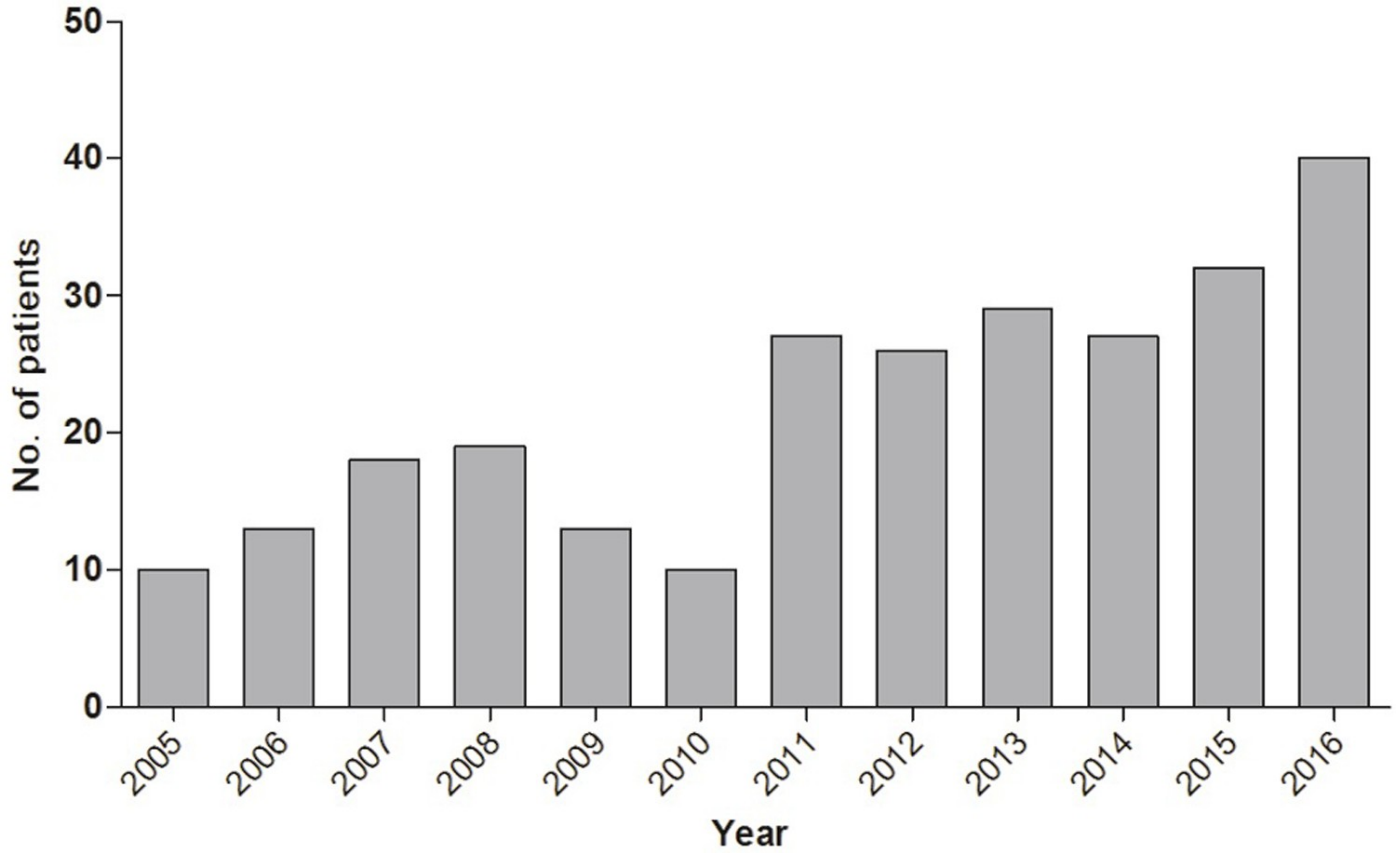
Annual change in number of advanced lung cancer patients admitted to medical intensive care unit.

There were 85 and 179 ICU admissions of advanced lung cancer patients in the 6-years pre-intensivist and 6-years post-intensivist system implementation, respectively. As shown in Fig 1, the number of advanced lung cancer patients admitted to the ICU increased each year. (p<0.004)

### Baseline characteristics of lung cancer patients at ICU admission

Demographic data and disease status of a total of 264 advanced lung cancer patients pre- and post-introduction of the intensivist system are shown in Table 1. The mean age of the patients was 67.2 years. Fifty-five (20.8%) patients were female. The proportion of patients with underlying pulmonary disease was statistically significantly higher in the pre-2011 groups (pre-2011: 44.7%, post-2011: 24.0%, p<0.001). There was no statistically significant difference, but after 2011, the number of patients who admitted ICU through ER decreased from 45.9% to 40.2%, and the number of patients admitted via hospital wards increased from 51.8% to 59.2%. Histopathologic types were as follows: non-small cell lung cancer (n = 222; 84.1%) including adenocarcinoma (n = 110; 41.7%), squamous cell carcinoma (n = 65; 24.6%), poorly differentiated carcinoma (n = 17; 6.4%), non-small cell carcinoma—not specified (n = 12; 4.6%), and others including neuroendocrine carcinoma and sarcomatoid carcinoma (n = 18; 6.8%); and small cell lung cancer (n = 32; 15.9%). Since 2011, the proportion of adenocarcinoma patients in the ICU increased from 32.9% to 45.8%, but the increase was not statistically significant (p = 0.050). Of the total number of patients, 195 (73.9%) patients were stage IV NSCLC, 27 (10.2%) stage IIIB, and 42 (15.0%) SCLC, ED. There were 58 (22.0%) newly diagnosed patients, 83 (31.4%) patients with disease controlled or in remission state, and 123 (46.6%) patients with disease recurrence or progression. The proportion of patients with advanced lung cancer admitted to the ICU after 2011 whose cancer status was controlled increased from 8.2% to 42.5%; this increase was statistically significant. Most patients (n = 218, 82.6%) underwent systemic chemotherapy prior to ICU admission. Thirty-eight (18.2%) patients underwent surgery, 39 (14.8%) underwent radiotherapy, and 41 (15.5%) underwent concurrent chemoradiation therapy. Besides the cancer control status, there were no significant differences in baseline characteristics between the two groups.

**Table 1:** Baseline characteristics of advanced lung cancer patients.

Variables	All patients (N = 264)	Pre 2011 (n = 85)	Post 2011(n = 179)	P value
Age (years old)	67.2 ± 9.8	66.1±10.1	67.7±9.6	0.217
Sex (male)	209 (79.2%)	70 (82.4%)	139 (77.7%)	0.380
Smoking history	190/259 (73.4%)	66/83(79.5%)	124/179 (69.3%)	0.124
Comorbidity^a^, n (%)				
Cardiovascular disease	32 (12.1%)	15 (17.7%)	17 (9.5%)	0.058
Cerebrovascular disease	13 (4.9%)	4 (4.7%)	9 (5.0%)	0.999
Pulmonary disease	81 (30.7%)	38 (44.7%)	43 (24.0%)	0.001
Diabetes mellitus	56 (21.2%)	21 (24.7%)	35 (19.6%)	0.339
Poor PS (ECOG PS 2–4)	179 (67.8%)	57 (67.1%)	122 (68.2%)	0.858
Admission route				0.211
Emergency department	111 (42.1%)	39 (45.9%)	72 (40.2%)	
Hospital ward	150 (56.8%)	44 (51.8%)	106 (59.2%)	
Surgical ICU	3 (1.1%)	2 (2.4%)	1 (0.6%)	
Histology, n(%)				0.050
ADC	110 (41.7%)	28 (32.9%)	82 (45.8%)	
SqCC	65 (24.6%)	19 (22.4%)	46 (25.7%)	
P/D carcinoma	17 (6.4%)	7 (8.2%)	10 (5.6%)	
NSCC	12 (4.6%)	8 (9.4%)	4 (2.2%)	
Others^b^	18 (6.8%)	8 (9.4%)	10 (5.6%)	
SCLC	42 (15.9%)	15 (17.7%)	27 (15.1%)	
Stage				0.846
IIIB	27 (10.2%)	8 (9.4%)	19 (10.6%)	
IV	195 (73.9%)	62 (72.9%)	133 (74.3%)	
Extensive disease	42 (15.9%)	15 (17.7%)	27 (15.1%)	
Cancer status in ICU, n (%)				<0.001
Controlled/remission	83 (31.4%)	7 (8.2%)	76 (42.5%)	
Newly diagnosed	58 (22.0%)	24 (28.2%)	34 (19.0%)	
Recur or progression	123 (46.6%)	54 (63.5%)	69 (38.6%)	
Previous treatment^c^, n(%)				
Surgery	48 (18.2%)	16 (18.8%)	32 (17.9%)	0.852
Chemotherapy	218 (82.6%)	75 (88.2%)	143 (79.9%)	0.095
Radiotherapy	39 (14.8%)	9 (10.6%)	30 (16.9%)	0.181
CCRT	41 (15.5%)	8 (9.4%)	33 (18.4%)	0.059
Severity at ICU admission				
SAPS II	55.3± 20.0	56.2± 18.4	54.9± 20.7	0.620
APACHE II	23.0± 9.8	23.9± 8.1	22.5± 10.4	0.278
SOFA score of 1^st^ ICU day	8.2± 4.5	7.9± 4.5	8.3± 4.6	0.554

^a^ Cardiovascular disease excluded essential hypertension, pulmonary disease included chronic obstructive pulmonary disease, interstitial lung disease, and bronchial asthma.

^b^ Others included large cell neuroendocrine carcinoma and sarcomatoid carcinoma.

^c^ Patients received multiple therapies, duplicated data.

Data are presented as n (%) or mean ± SD.

PS = performance status ECOG = The Eastern Cooperative Oncology Group ADC = adenocarcinoma SqCC = squamous cell carcinoma P/D carcinoma = poorly differentiated carcinoma NSCC = non-small cell cancer SCLC = small cell lung cancer ICU = intensive care unit CCRT = concurrent chemoradiation therapy SAPS II = Simplified Acute Physiology Score II APACHE II = Acute Physiology and Chronic Health Evaluation II SOFA score = Sequential Organ Failure Assessment score.

### Reason for ICU admission

Table 2 presents the reasons for ICU admission of the patients. As per the first classification based on the oncologist’s perspective, 91 (34.5%) patients experienced cancer related events, such as obstructive pneumonia and malignant airway obstruction; 86 (32.6%) patients were admitted to the ICU for treatment related events including radiation pneumonitis, chemotherapy-induced lung toxicity, and neutropenic infection; 50 (18.9%) patients experienced infections, not clearly related to malignancy or treatment; and 18 patients (6.8%) were admitted to the ICU for causes including opioid toxicity, contrast induced anaphylaxis, gastrointestinal bleeding, transfusion-related acute lung injury, and cardiopulmonary arrest due to unknown causes. The proportion of patients admitted due to treatment-related adverse events was reduced in patients admitted after 2011 (pre-2011: 60.0%, post-2011: 19.6%, p<0.001).

**Table 2:** Cause of intensive care unit (ICU) admission of advanced lung cancer, compared intensivist’s vs. oncologist’s perspective.

	All patients (N = 264)	Pre 2011 (n = 85)	Post 2011 (n = 179)	P value
Oncologist’s perspective, n (%)				<0.001
Cancer related events^a^	91 (34.5%)	28 (32.9%)	63 (35.2%)	
Treatment related events^b^	86 (32.6%)	51 (60.0%)	35 (19.6%)	
Infection, not clearly related to cancer or treatment^c^	50 (18.9%)	2 (2.4%)	48 (26.8%)	
Comorbidity related events^d^	19 (7.2%)	4 (4.7%)	15 (8.4%)	
Others^e^	18 (6.8%)	0	18 (10.1%)	
Intensivist’s perspective, n (%)				0.525
Respiratory failure	205 (77.7%)	70 (82.4%)	135 (75.4%)	
Neurologic defect	12 (4.6%)	4 (4.7%)	8 (4.5%)	
Kidney failure	6 (2.3%)	2 (2.4%)	4 (2.2%)	
Cardiac problem	32 (12.1%)	6 (7.1%)	26 (14.5%)	
Others^f^	9 (3.4%)	3 (3.5%)	6 (3.4%)	

Data are presented as n (%).

^a^ Cancer related events, included obstructive pneumonia, respiratory failure due to lung involvement of cancer.

^b^ Treatment-related events, including radiation pneumonitis, chemotherapy-induced lung toxicity, neutropenic infection.

^c^ Infection, not clearly related to cancer or treatment included pneumonia.

^d^ Comorbidity related events included underlying pulmonary disease and cardiovascular disease related acute events.

^e^ Others included opioid toxicity, unknown cause of cardiopulmonary arrest, contrast induced anaphylaxis, gastrointestinal bleeding, unknown cause of acute decompensated heart failure, and transfusion-related acute lung injury (TRALI).

^f^ Others included gastrointestinal bleeding, unknown cause of cardiopulmonary arrest, contrast induced anaphylaxis.

As per the second classification, we categorized the reason for ICU admission based on major organ failure and requirement of intensive care. Respiratory failure requiring oxygen therapy was seen in 205 (77.7%) patients, 12 (4.6%) patients had neurologic defects requiring intensive monitoring, 6 (2.3%) patients had kidney failure requiring CRRT, 32 (12.1%) patients were admitted for cardiac problems, such as cardiac tamponade and acute coronary syndrome, and 9 (3.4%) patients were admitted for other causes, such as massive bleeding and anaphylaxis. After 2011, there were a greater proportion of patients with ICU admissions related to cardiac problems (pre-2011: 7.1%, post-2011: 14.5%, p < 0.001).

### Comparison of clinical outcomes of advanced lung cancer patients admitted to the ICU before and after introduction of the intensivist system

Table 3 shows the clinical outcomes of the ICU admitted patients. Overall 30-day ICU mortality rate was 41.3% (n = 109) and hospital mortality rate was 71.2% (n = 188). ICU LOS was 10.3±12.0 days and hospital LOS was 26.7±27.3 days. After implementation of the intensivist system in the ICU, there was a statistically significant decrease in hospital mortality (pre-2011: 82.4%, post- 2011: 65.9%, p = 0.006), the ICU LOS (pre-2011: 14.5 ± 16.5 days, post-2011: 8.3±6.6 days, p<0.001), and the hospital LOS (pre-2011; 36.6 ± 37.2 days, post-2011; 22.0 ± 19.6 days, p<0.001). The performance status of ICU survivors improved after 2011 (mean ECOG PS pre-2011; 3.7 ± 0.7, post-2011; 3.3 ± 0.9, p = 0.022), but there was no statistically significant improvement in performance status of hospital survivors (pre-2011; 3.2 ± 0.8, post-2011; 3.0 ± 1.1, p = 0.545). The crude 30-day ICU mortality decreased from 43.5% to 40.2% after 2011, but the decrease was not statistically significant (p = 0.610). In this study, the proportion of patients who have completed DNR during ICU periods since 2011 has been increased from 57.1% to 61.1%. (p = 0.575) (data included in S3 Appendix).

**Table 3:** Clinical outcomes according to presence of intensivist.

	All patients (N = 264)	Pre 2011 (n = 85)	Post 2011 (n = 179)	P value
30-day ICU mortality, n (%)	109 (41.3%)	37 (43.5%)	72 (40.2%)	0.610
Hospital mortality, n (%)	188 (71.2%)	70 (82.4%)	118 (65.9%)	0.006
ICU LOS (d)	10.3 ± 12.0	14.5 ± 16.5	8.3 ±8.6	<0.001
Hospital LOS (d)	26.7 ± 27.3	36.6 ±37.2	22.0 ±19.6	<0.001
ECOG PS at ICU discharge^a^	3.4 ± 0.9 (n = 143)	3.7 ± 0.7 (n = 39)	3.3 ± 0.9 (n = 104)	0.022
ECOG PS at hospital discharge^a^	3.1 ± 1.0 (n = 76)	3.2 ± 0.8 (n = 15)	3.0 ± 1.1 (n = 61)	0.545

Data are presented as n (%) or mean ± SD.

^a^Statistical analysis excluded mortality cases

ICU = intensive care unit LOS = length of stay ECOG = The Eastern Cooperative Oncology Group PS = performance status

Subgroup analysis was performed to determine which patients benefited from the intensivist system. (S1 Table) We analyzed patients with the following medical conditions: acute respiratory failure, sepsis, pneumonia, and neutropenic infection. Patients with acute respiratory failure (n = 212) showed statistically significant decrease in ICU LOS and hospital LOS (p < 0.001).

### Logistic regression analysis of 30-day ICU mortality and hospital mortality

We first performed a univariate logistic regression analysis to determine which factors influenced the 30-day ICU mortality and hospital mortality (S2 Table). Non-survivors had higher SAPS II, APACHE II, and SOFA scores on the 1^st^ day in ICU and lower P/F ratios, absolute platelet count, hemoglobin, and serum albumin than survivors. In the ICU, a higher proportion of non-survivors received treatment with high flow nasal cannula, MV, vasoactive agents, and CRRT. Based on the results of univariate analysis, we designed a multivariate analysis model to assess which factors affected the clinical outcome (Table 4). In this logistic regression model, patients’ sex, age, performance status, severity at ICU admission, cancer control status were included. Multivariate analysis adjusted for age, sex, performance status, cancer control status and SAPS II score showed that admissions after 2011 were independently associated with decreased in-hospital mortality (Odds ratio (OR) 0.40, 95% confidence interval (CI): 0.21–0.77, p = 0.006).

**Table 4:** Logistic regression analysis of 30-day intensive care unit (ICU) mortality and hospital mortality determinants.

	30-day ICU mortality				In hospital mortality			
	Univariate analysis		Multivariate analysis		Univariate analysis		Multivariate analysis	
Variables	OR (95% CI)	P-value	OR (95% CI)	P-value	OR (95% CI)	P-value	OR (95% CI)	P-value
Sex (Male)	1.18 (0.64–2.17)	0.599	1.13 (0.59–2.17)	0.703	1.27 (0.67–2.40)	0.469	1.34 (0.66–2.70)	0.414
Age≥ 65yr	1.29 (0.76–2.18)	0.350	1.11(0.63–1.96)	0.710	1.31 (0.74–2.29)	0.348	1.21(0.65–2.26)	0.541
Poor PS (ECOG PS ≥ 2)	1.68 (0.98–2.89)	0.059	1.55 (0.987–2.877)	0.135	2.55 (1.46–4.45)	0.001	2.43 (1.33–4.44)	0.004
SAPS II score	1.03 (1.01–1.04)	<0.001	1.03 (1.01–1.04)	<0.001	1.02 (1.00–1.03)	0.013	1.01 (0.99–1.03)	0.063
Admitted after 2011	0.87 (0.52–1.47)	0.610	0.96 (0.53–1.572)	0.886	0.42 (0.22–0.78)	0.007	0.40 (0.20–0.81)	0.011
Cancer status								
Controlled/remission	reference		reference		reference		reference	
Newly diagnosed	0.75 (0.37–1.54)	0.439	0.77 (0.36–1.67)	0.515	0.64 (0.32–1.27)	0.202	0.53 (0.24–1.13)	0.101
Recur or progression	1.60 (0.91–2.82)	0.106	1.52 (0.81–2.86)	0.189	1.99 (1.05–3.77)	0.035	1.45 (0.73–2.90)	0.288

Data are presented as n (%) or mean ± SD

ICU = intensive care unit OR = odds ratio CI = confidence interval PS = performance status ECOG = the Eastern Cooperative Oncology Group SAPS II = Simplified Acute Physiology Score II

### The change of clinical outcomes of patients with advanced lung cancer in ICU after intensivist system implementation

**Fig 2:**
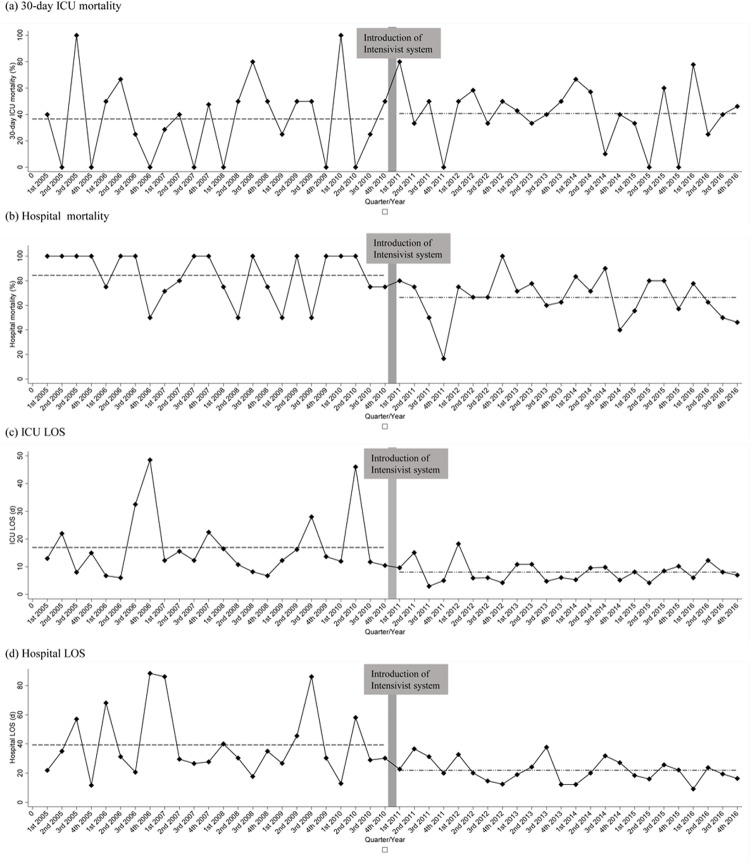
Run chart of clinical outcomes of advanced lung cancer patients admitted medical intensive care unit. (a) 30-day ICU mortality (b) Hospital mortality (c) ICU LOS (d) Hospital LOS ICU = intensive care unit LOS = length of stay.

Since the implementation of the intensivist system, the quarterly 30-day ICU mortality, hospital mortality, ICU LOS, and hospital LOS of advanced lung cancer patients showed a gradual decline (Fig 2). Statistical tests using the ARIMA model showed that hospital mortality (p = 0.001) and hospital LOS (p<0.001) were significantly reduced after introduction of the intensivist system. The overall ICU mortality was significantly reduced in the ARIMA model (p = 0.017), but there was no significant difference in the 30-day ICU mortality (p = 0.450) (S3 Table).

## Discussion

Our study demonstrated that the implementation of the intensivist system reduced hospital mortality, ICU LOS, and hospital LOS in advanced lung cancer patients. These results were consistent in the time series analyses besides the crude analysis comparing pre- and post-2011. Admission of patients with advanced lung cancer increased steadily over the years and there was no significant difference in severity scores (SAPS II, APACHE II, and SOFA scores on day 1). Survival rate improved after 2011, that is, after introduction of the intensivist.

Intensivist staffing is currently the standard protocol in ICU care; however, little research has been conducted on whether this system improves clinical outcomes in advanced cancer patients. Recent studies with regard to critical care in lung cancer patients were focused on admission criteria [14,26] and prognostic factors [27, 28]. A retrospective study published in 2016 reported that collaboration between oncologists and intensivists, presence of clinical pharmacists in the ICU, and organized protocols were associated with lower mortality in cancer patients [29]. However, only 20% of the ICUs included in this study had board-certified intensivists [29]. In addition, according to the study published in 2014, only 29.3% of Korean ICU had intensive specialists [30]. To the best of our knowledge, this is the first study to demonstrate the association of the pulmonary intensivist system with improved clinical outcomes in advanced lung cancer patients.

A previous study from our center classified the reasons for ICU admission from the oncologist’s point of view [14]. Whereas, this study classified them from the perspective of intensivists. The most common reason for ICU admission was respiratory failure (78% of patients), and patients were supported with mechanical ventilation, non-invasive ventilation, oxygen support, or even extracorporeal membrane oxygenation. The second common reason for ICU admission was cardiac problems, and patient interventions in these cases included percutaneous catheter drainage and percutaneous coronary interventions. The proportion of patients admitted to ICU due to cardiovascular diseases was higher in the post-2011 group, probably because the intensive monitoring system was introduced before and after the high-risk interventions described above.

In this study, it was observed that 73.1% of patients received MV. One study using the Medicare registry [3] reported that 21% patients with lung cancer admitted to the ICU received invasive ventilation, and a multinational study conducted in Europe and South America found that 55% patients received ventilatory support [27]. In our study, ICU LOS decreased by 6.4 days and hospital LOS decreased by 13.3 days in patients with acute respiratory failure, after the intensivist system implementation. The high proportion of patients requiring mechanical ventilation may be attributed to the fact that the pulmonary intensivists provide organized treatments, such as active use of noninvasive ventilation, implementing ventilator weaning protocols, and treating ventilator-associated pneumonia. Systematic treatment of acute respiratory distress syndrome (ARDS) based on the guideline [31] also contributed to the improvement of clinical outcomes of advanced lung cancer patients.

The ongoing controversy over ICU outcomes of cancer patients is mainly because of the heterogeneity of previous studies and the extremely low survival rates of advanced cancer patients. Specifically, recurrent/progressive disease status and treatment limitation were the main determinants of 30-day ICU mortality [27]. The prognosis of unresectable lung cancer is expected to improve with the introduction of target therapy and immunotherapy, suggesting that the indications for ICU admission may be extended for patients with progressive disease [13]. In South Korea, health insurance beneficiaries are allowed to prescribe immune checkpoint inhibitor after 2017. The rapid increase of prescription of the immune checkpoint inhibitors will improve the survival rate of lung cancer patients, and ICU care of critically ill lung cancer patients will need further study in the future. We suggest that the system of dedicated intensivists can be helpful in improving the clinical outcome in these patients.

There are several limitations in this study. First, this study was performed by retrospective review of records from a single center. Second, introduction and operation of the intensivist system have not been quantified. Not only the intensivist system but also the comprehensive care may have contributed to clinical outcome improvement. Third, patients were categorized based on single organ failure leading to critical illness, but many cancer patients have multiple organ failure leading to critical condition. Fourth, defect in surveillance of satisfaction and quality of life (qOL) of patients is one of limitations of our study. Fifth, advances in medical technology and new therapies have been introduced in recent years, which could have contributed to the improved prognosis after 2011. Most of the patients included in this study were patients after 2011, and selection bias is a concern because they would benefit from this advanced medicine. The patients with adenocarcinoma, have benefits with targeted therapies increased since 2011. Finally, it should be recalled that there has been a change in the composition of the patient population since 2011. In addition to changes in the histologic diagnosis of the patients, changes in admission route after 2011 may also have been associated with an improvement in clinical outcomes. In order to account for this, a time-series analysis was conducted, but the improvement of the result cannot be completely ruled out in results of this study.

Based on clinical judgement of resident or attending physicians, an withholding or withdrawal of life-sustaining treatment of the patient was made when the patient had entered an irreversible end of life. This study covers the period before the Law of withholding or withdrawal of life-sustaining treatment was enacted in South Korea, 2017, and ICU LOS of advanced lung cancer patients will continue to be shortened in the future.

Although there are not many studies on ICU care in patients with advanced lung cancer, the number of surviving patients is continuously increasing due to the development of treatment. A prospective study should be conducted to accurately demonstrate that intensivist care is more effective for the ICU care of lung cancer patients. Stepped-wedge, cluster-randomized trial design adopted in PARTNER trial, published June 2018, may be helpful in proving this hypothesis [32].

In conclusion, the dedicated intensivist system contributed to improving the hospital survival of critical patients with advanced lung cancer and shortening ICU and hospital LOS. These improvements seem to have a correlation to the main reason for ICU admission in advanced lung cancer patients, which is respiratory failure. Further studies are needed to confirm these findings and studies on optimal selection criteria for ICU admission should be performed concurrently.

## Supporting information

S1 Table Clinical outcomes according to presence of intensivist in subgroups. (DOCX)

S2 Table Univariate logistic regression analysis of intensive care unit (ICU) mortality. (DOCX)

S3 Table Autoregressive integrated moving average (ARIMA) model parameter estimate of the impact of the comprehensive care including intensivist system on clinical outcomes of advanced lung cancer patients. (DOCX)

S1 Appendix Protocol of acute respiratory distress syndrome management (2016 version). (PDF)

S2 Appendix Protocol of weaning from mechanical ventilation. (DOCX)

S3 Appendix anonymized dataset of the study. (XLSX)
