# Supplementary material for: Effect of intensivist involvement on clinical outcomes in patients with advanced lung cancer admitted to the intensive care unit
Source: PLoS One. 2019 Feb 13;14(2):e0210951. doi: 10.1371/journal.pone.0210951 (PMC6373899; doi:10.1371/journal.pone.0210951)
Supplement: S1 Table — (DOCX) [file pone.0210951.s001.docx]

**Table S1. Clinical outcomes according to presence of intensivist in subgroups**

| **Clinical outcome** | **Subgroup** | **Pre-2011** | **Post-2011** | **P-value** |
| --- | --- | --- | --- | --- |
|  | **Acute respiratory failure (n=212)** | Pre-2011 (n=75) | Post-2011 (n=137) |  |
| 30-day ICU mortality (%) | 99 (46.7%) | 36 (48.0%) | 63 (46.0%) | 0.779 |
| Hospital mortality (%) | 161 (75.9%) | 62 (82.7%) | 99 (72.3%) | 0.090 |
| ICU LOS (d) | 11.7±12.5 | 15.8 ± 17.1 | 9.4 ±8.3 | <0.001 |
| Hospital LOS (d) | 28.2±28.9 | 37.0 ±38.6 | 23.3 ±20.4 | <0.001 |
|  | **Sepsis (n=69)** | Pre-2011 (n=22) | Post-2011 (n=47) |  |
| 30-day ICU mortality (%) | 41 (59.4%) | 14 (63.6%) | 27 (57.5%) | 0.626 |
| Hospital mortality (%) | 56 (81.2%) | 16 (72.7%) | 40(85.1%) | 0.322 |
| ICU LOS (d) | 10.3±10.6 | 9.1±11.5 | 10.9±10.3 | 0.515 |
| Hospital LOS (d) | 24.1±25.4 | 24.3±34.7 | 24.0±20.1 | 0.970 |
|  | **Pneumonia (n=118)** | Pre-2011 (n=45) | Post-2011 (n=73) |  |
| 30-day ICU mortality (%) | 59 (50%) | 23 (51.1%) | 36 (49.3%) | 0.850 |
| Hospital mortality (%) | 98 (83.1%) | 39 (86.7%) | 59 (80.8%) | 0.411 |
| ICU LOS (d) | 13.0±13.4 | 15.3±17.0 | 11.5±10.5 | 0.135 |
| Hospital LOS (d) | 28.4±27.8 | 31.5±34.6 | 26.5±22.8 | 0.344 |
|  | **Neutropenic infection (n=30)** | Pre-2011 (n=10) | Post-2011 (n=20) |  |
| 30-day ICU mortality (%) | 19 (63.3%) | 5 (50.0%) | 14 (70.0%) | 0.425 |
| Hospital mortality (%) | 23 (76.7%) | 6 (60.0%) | 17 (85.0%) | 0.181 |
| ICU LOS (d) | 11.2±13.4 | 10.8±15.3 | 11.5±12.8 | 0.903 |
| Hospital LOS (d) | 22.8±29.7 | 28.1±46.1 | 20.2±17.9 | 0.502 |

ICU = intensive care unit, LOS = length of stay
