# Supplementary material for: Effect of intensivist involvement on clinical outcomes in patients with advanced lung cancer admitted to the intensive care unit
Source: PLoS One. 2019 Feb 13;14(2):e0210951. doi: 10.1371/journal.pone.0210951 (PMC6373899; doi:10.1371/journal.pone.0210951)
Supplement: S2 Table — (DOCX) [file pone.0210951.s002.docx]

**Table S2. Univariate logistic regression analysis of intensive care unit (ICU) mortality**

| **Variables** | **All patients (n = 264)** | **30-day ICU survivor (n=155)** | **30-day ICU non-survivor (n=109)** | **P value** |
| --- | --- | --- | --- | --- |
| Age (years old) | 67.2 ± 9.8 | 66.3 ± 9.9 | 68.5 ± 9.4 | 0.068 |
| Sex (male) | 209 (79.2%) | 121 (78.1%) | 88 (80.7%) | 0.599 |
| Comorbidity, n (%) |  |  |  |  |
| Cardiovascular disease | 32 (12.1%) | 19 (12.3%) | 13 (11.9%) | 0.935 |
| Cerebrovascular disease | 13 (4.9%) | 8 (5.2%) | 5 (4.6%) | 0.832 |
| Pulmonary disease | 81 (30.7%) | 30 (27.5%) | 51 (32.9%) | 0.351 |
| Diabetes mellitus | 56 (21.2%) | 34 (21.9%) | 22 (20.2%) | 0.732 |
| Histology, n (%) |  |  |  | 0.290 |
| ADC | 110 (41.7%) | 70 (45.2%) | 40 (36.7%) |  |
| SqCC | 65 (24.6%) | 41 (26.5%) | 24 (22.0%) |  |
| P/D carcinoma | 17 (6.5%) | 8 (5.2%) | 9 (8.3%) |  |
| NSCC | 12 (4.6%) | 6 (3.9%) | 6 (5.5%) |  |
| Others^a^ | 18 (6.8%) | 7 (4.5%) | 11 (10.1%) |  |
| SCLC | 42 (15.9%) | 23 (14.8%) | 19 (17.4%) |  |
| Admission route |  |  |  | 0.007 |
| Emergency department | 111 (42.1%) | 54 (34.8%) | 57 (52.3%) |  |
| Hospital ward | 160 (56.8%) | 98 (63.2%) | 52 (47.7%) |  |
| Surgical ICU | 3 (1.1%) | 3 (1.9%) | 0 (0%) |  |
| Severity at ICU admission |  |  |  |  |
| SAPS II | 55.3 ± 20.0 | 51.1 ± 19.0 | 61.4 ± 19.8 | <0.001 |
| APACHE II | 23.0 ± 9.8 | 20.4 ± 8.6 | 26.6 ± 10.1 | <0.001 |
| SOFA score of 1^st^ ICU day | 8.2 ± 4.5 | 7.0 ± 4.4 | 9.9 ± 4.3 | <0.001 |
| Results of blood chemistry |  |  |  |  |
| PaO2/FiO2 | 178.3 ± 112.5 | 197.0 ± 120.5 | 152.0 ± 94.7 | 0.001 |
| ANC (k) | 11.2 ± 9.9 | 11.5 ± 9.4 | 10.8 ± 10.5 | 0.544 |
| Platelet (k) | 205.1 ± 125.2 | 228.7 ± 120.3 | 171.6 ± 124.9 | <0.001 |
| Hemoglobin (g/dL) | 10.6 ± 2.0 | 10.8 ± 1.8 | 10.2 ± 2.2 | 0.012 |
| Serum albumin (g/dL) | 2.9 ± 0.6 | 2.9 ± 0.6 | 2.7 ± 0.5 | 0.001 |
| Treatment during ICU stay, n (%) | | | | |
| NIV | 12 (4.6%) | 9 (5.8%) | 3 (2.8%) | 0.370 |
| HFNC | 33 (12.5%) | 27 (17.4%) | 6 (5.5%) | 0.004 |
| MV | 193 (73.1%) | 89 (57.4%) | 104 (95.4%) | <0.001 |
| Vasoactive agents | 158 (59.9%) | 75 (48.4%) | 83 (76.2%) | <0.001 |
| CRRT | 30 (11.4%) | 9 (5.8%) | 21 (19.3%) | 0.001 |
| Chemotherapy in ICU | 9 (3.4%) | 6 (3.9%) | 3 (2.8%) | 0.740 |
| Intensivist referral, n (%) | 133 (50.4%) | 76 (49.0%) | 57 (52.3%) | 0.602 |

ADC = adenocarcinoma SqCC = squamous cell carcinoma P/D carcinoma = poorly differentiated carcinoma NSCC = non-small cell cancer SCLC = small cell lung cancer ICU = intensive care unit SAPS II = Simplified Acute Physiology Score II APACHE II =Acute Physiology and Chronic Health Evaluation II SOFA score = Sequential Organ Failure Assessent score ANC = absolute neutrophil count NIV = noninvasive ventilation HFNC = high flow nasal cannula MV = mechanical ventilation CRRT = continuous renal replacement therapy.

Data are presented as n (%) or mean ± SD.

^a^Others included large cell neuroendocrine carcinoma and sarcomatoid carcinoma.
