# Supplementary material for: Effect of intensivist involvement on clinical outcomes in patients with advanced lung cancer admitted to the intensive care unit
Source: PLoS One. 2019 Feb 13;14(2):e0210951. doi: 10.1371/journal.pone.0210951 (PMC6373899; doi:10.1371/journal.pone.0210951)
Supplement: S3 Table — (DOCX) [file pone.0210951.s003.docx]

**Table S3. Autoregressive integrated moving average (ARIMA) model parameter estimate of the impact of the comprehensive care including intensivist system on clinical outcomes of advanced lung cancer patients**

|  | **Estimate** | **SE** | **P-value** |
| --- | --- | --- | --- |
| 30-day ICU mortality | 3.28 | 4.34 | 0.450 |
| Overall ICU mortality | -11.91 | s5.00 | 0.017 |
| Hospital mortality | -17.95 | 5.31 | 0.001 |
| Mean ICU LOS | -0.09 | 0.71 | 0.900 |
| Mean hospital LOS | -0.50 | 0.13 | <0.001 |

*SE* standard error
