# Supplementary material for: Effect of intensivist involvement on clinical outcomes in patients with advanced lung cancer admitted to the intensive care unit
Source: PLoS One. 2019 Feb 13;14(2):e0210951. doi: 10.1371/journal.pone.0210951 (PMC6373899; doi:10.1371/journal.pone.0210951)
Supplement: S1 Appendix — (PDF) [file pone.0210951.s004.pdf]

Edited by lungdrcho, 2016.11. ver 1.0

SNU ARDS Protocol

[Within 1 week, P/F≤300 with CPAP or PEEP 5 cmH_2O, Bilateral opacities-not fully explained by effusions, lobar/lung collapse, or nodules, Not fully explained by cardiac failure or fluid overload]

Part I: MV strategies

1. Calculate predicted body weight (PBW): **M** 50 + (0.91) [height (cm) – 152.4] **F** 45.5 + (0.91) [height (cm) – 152.4]
2. Select any ventilator mode (If possible, start with VC mode to measure initial Pplat and C_{RS} by inspiration hold with inspiratory pause 0.5s).
3. Set ventilator settings to achieve initial V_T = 8 ml/kg PBW.
4. Reduce V_T by 1 ml/kg at intervals ≤ 2 hours until **V_T <= 6 ml/kg PBW**.
5. Set initial RR to approximate baseline minute ventilation (not > 35 bpm).
6. Adjust V_T and RR to achieve pH and plateau pressure goals below.
7. Target Oxygenation: **PaO_2 55-80 mmHg or SpO_2 88-95%**
   (Use a minimum PEEP of 5 cmH_2O. Consider use of incremental FiO_2/PEEP combinations.)
8. Target Driving Pressure (**ΔP = Pplat – PEEP**): **15 cmH_2O**
   (Check Pplat at least q8h and after each change in PEEP or V_T)
9. Target **pH 7.30-7.45** (If acidosis, increase RR up to max 35; If alkalosis decrease RR if possible)
10. I:E ratio: duration of inspiration <= duration of expiration

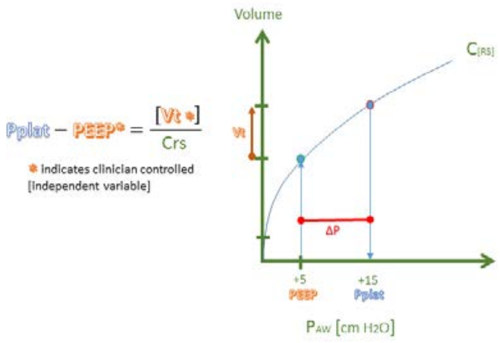

Goal of parameters	ARDS	
Severity	Mild (200<P/F ratio<=300)	Moderate-Severe (P/F ratio<=200)
Tidal volume	6–8 ml/kg predicted body weight	6–8 ml/kg predicted body weight
Level of PEEP	5 – 10 cmH_2O	≥ 10 cmH_2O
Driving Pressure (or Pplat)	<15 cmH_2O (<30 cmH_2O)	<15 cm H_2O (<30 cmH_2O)

Edited by lungdrcho, 2016.11. ver 1.0

Part II: Non-MV strategies

<KATRD/KSCCM 2016 CPG guideline>

Recommendations	Level of evidence	ARDS		Non-ARDS	
		Pros	Cons	Pros	Cons
1	A	Low tidal volume ventilation	Inhaled nitric oxide	-	-
	B	Prone position<br>Light sedation	HFOV	Low tidal volume ventilation<br>Light sedation	-
	C	-	-	-	-
2	A	-	-	Early tracheostomy (only limited cases)	-
	B	High PEEP (if P/F≤200)<br>Recruitment maneuver<br>Neuromuscular blockage	Systemic steroids	Lung protective ventilation strategy (intraoperative)	-
	C	ECMO	-	-	

ARDS: acute respiratory distress syndrome; PEEP: positive end-expiratory pressure; ECMO: extracorporeal membrane oxygenation; HFOV: high-frequency oscillatory ventilation.

1. Prone position
   A. Severe ARDS: P/F ratio<150 mmHg, with FiO_2 >0.6, PEEP> 5 cmH_2O and V_T~6 ml/kg PBW
   B. At least for 16hrs, follow your own hospital healthcare providers' situation
2. ECMO
   A. No contraindication including non-protective MV over 7 days, Consider candidate for lung transplantation
   B. After Part I, i) uncontrollable acidosis: pH<7.15 or ii) unacceptable oxygenation: P/F ratio <50 with FiO_2>0.9 for >3 hrs
   C. Consider hemodynamics, decide whether VV-ECMO vs. VA-ECMO vs. $ECCO_2R$.
3. Discuss with attending physician when considering use of iNO, systemic steroid, or other experimental treatments.

Will be updated every year by SNU-CCM
