# Supplementary material for: Effect of intensivist involvement on clinical outcomes in patients with advanced lung cancer admitted to the intensive care unit
Source: PLoS One. 2019 Feb 13;14(2):e0210951. doi: 10.1371/journal.pone.0210951 (PMC6373899; doi:10.1371/journal.pone.0210951)
Supplement: S2 Appendix — (DOCX) [file pone.0210951.s005.docx]

SNUBH Weaning and Extubation Protocol

1. **Indication:**

| Considerations for assessing readiness for weaning | |
| --- | --- |
| Clinical assessment | Adequate cough  Absence of excessive tracheobronchial secretion  Resolution of disease acute phase for which the patient was intubated |
| Objective measurements | *Clinical stability*  Stable cardiovascular status (i.e. HR ≤ 120/min, SBP 90~140 mmHg, no or minimal vasopressors: dopa 5mcg/kg/min, levo 0.05mcg/kg/min)  Stable metabolic status  *Adequate oxygenation*  SaO_2_ >90% on FiO_2_ ≤0.4 (or PF ratio > 200)  PEEP ≤5 cmH_2_O  *Adequate pulmonary function*  RR <35/min  MIP ≤-20 cmH_2_O  V_T_ >5 mL/kg  V_E_ <10~15 L/min  RSBI [RR (in breaths/min) / V_T_ (in liters)] <105  No significant respiratory acidosis  *Adequate mentation*  No sedation or adequate mentation on sedation |

1. **Goal:** There is the stable status in subjective comfort, including no physiologic increase of heart rate, blood pressure, or respiratory rate, after release from ventilatory support, and no hypoxemia or respiratory acidosis at 50% of inspired oxygen concentration.
2. **Step by step approach:**

| Daily Assessment for SBT | |
| --- | --- |
| □ Clinical stability  □ Adequate mentation  □ Adequate oxygenation  - SaO_2_ >90% on FiO_2_ ≤0.4  (or PF ratio > 200)  - PEEP ≤5 cmH_2_O | □ Adequate pulmonary function  - RR <35/min  - MIP ≤-20 cmH_2_O  - V_T_ >5 mL/kg  - V_E_ <10~15 L/min  - no significant respiratory acidosis |

Meets All Readiness Criteria

Begin CPAP of 0 cmH_2_O for 3 min for RSBI

Yes No

Resume appropriate ventilator settings, allow patients to rest for 24 hrs

RSBI <105 RSBI ≥105

Spontaneous breathing trial (SBT) for 30~120 min

: T-piece trial with 10 L/min of O_2_ at FiO_2_ 0.4

No

Successful SBT

Yes

* Cuff-leak test

Trial of Extubation

- * Cuff leak test (optional): decrease in exhaled volume after cuff deflation of < 110mL or 25% change

Average of 3 values on 6 consecutive breaths measured during volume-cycled ventilation

| Criteria for SBT failure | |
| --- | --- |
| Clinical assessment | Objective measurements |
| Agitation and anxiety  Depressed mental status  Diaphoresis  Cyanosis  Evidence of increasing effort  Increased accessory muscle activity  Facial signs of distress  Dyspnea | PaO_2_ <60 mmHg or SaO_2_ <90% on FiO_2_ ≥0.4  PaCO_2_ >45 mmHg or an increase in ≥20% from pre-SBT  pH <7.32 or a decrease in pH ≥0.07  RR >35/min or increased by ≥50%  HR >140/min or increased by ≥20%  SBP >180 mmHg or increased by ≥20%  SBP <90 mmHg  Cardiac arrhythmias |

1. **References:**
2. Boles J-M, Bion J, Connors a, et al. Weaning from mechanical ventilation. *The European Respiratory Journal*. 2007;29(5):1033–56.
3. El-Khatib MF, Bou-Khalil P. Clinical review: liberation from mechanical ventilation. *Critical Care*. 2008;12(4):221.
